# Multinomial inference on distributed responses in SPM

**DOI:** 10.1016/j.neuroimage.2010.05.076

**Published:** 2010-10-15

**Authors:** J.R. Chumbley, G. Flandin, M.L. Seghier, K.J. Friston

**Affiliations:** The Wellcome Trust Centre for Neuroimaging, UCL, UK

## Abstract

In this work, we propose statistical methods to perform inference on the spatial distribution of topological features (e.g. maxima or clusters) in statistical parametric maps (SPMs). This contrasts with local inference on the features *per se* (e.g., height or extent), which is well-studied (e.g. Friston et al., 1991, 1994; Worsley et al., 1992, 2003, 2004). We present a Bayesian approach to detecting experimentally-induced patterns of distributed responses in SPMs with anisotropic, non-stationary noise and arbitrary geometry. We extend the framework to accommodate fixed- and random-effects analyses at the within and between-subject levels respectively. We illustrate the method by characterising the anatomy of language at different scales of functional segregation.

## Research Highlights

►Given a fixed partition of an SPM (eg anatomical altas/independent ROIs), which regions are relatively active?►This method identifies 'active' regions as containing more events (e.g. blobs) than expected by chance►The method is sensitive to different features of an SPM, relative to conventional analyses

## Introduction

The paradigm of functional segregation in cognitive neuroscience entails differential engagement of distinct brain regions. An example is the famously problematic hypothesis that region Q is engaged and region R is not activated; i.e. functional specialisation or segregation. This segregation of specialised or functionally selective responses in the brain requires that responses are specific to certain brain regions. We will refer to this as ‘regional specificity’. Mass-univariate approaches (like SPM) cannot address regional specificity, because one can never infer R is not activated (i.e., accept the null). There is a general paucity of methods for addressing hypotheses about the specificity of distributed effects in neuroimaging. Historically, the SPM school calls on the so-called ‘topological’ rather than spatial inference, which considers topological features of statistical parametric maps like maxima or regional excursion sets, as opposed to individual voxels (e.g., Chumbley et al., 2010). The present work is inherently multivariate in that it harvests statistics from different parts of the brain, and can provide an answer to the question: is region Q more engaged than region R, in terms of ‘event’ density, where ‘events’ are general data-features whose spatial distribution can be assumed under the null.

Clearly, to make an inference that one part of the brain responds more than another part, we have to consider regional responses. This takes us out of the mass-univariate (voxel-based) inference framework used by SPM and obliges us to define the regions entailed by relative regional effects. This definition relaxes the dependence on spatial smoothing that is an integral part of most conventional SPM analyses: to the extent that experimentally-induced responses are conserved spatially over subjects, they can combine to give significant group effects in between-subject SPM analyses. One motivation for smoothing is to ensure responses from each subject overlap by smearing them. This requires effects in different subjects to be close, relative to the scale of the smoothing kernel. In turn, this induces the problem of optimising the scale of the kernel and leads to the notion of scale-space searches (Poline and Mazoyer, 1994a,b; Worsley et al., 1996). Here, we eschew this problem by only requiring that responses fall predominantly within pre-defined regions (e.g. Brodmann's regions). Regional summaries of per subject ‘events’ can then be assessed in relation to each other to provide inference at the spatial scale of the parcellation scheme chosen.

We focus on the special case when ‘events’ are maxima/peaks of a real-valued SPM, resulting from the estimation of a general linear model (GLM) point-wise over the brain. Assuming that randomness in the component fields (i.e. error fields) of the GLM take the form of a Gaussian-field, closed-form solutions for the rate of local maxima in the SPM exceeding some arbitrary height have been derived using random field theory (RFT: e.g. Friston et al., 1991, 1994; Worsley et al., 1992, Worsley et al., 2004). This number has been shown, in the limit of high thresholds, to have a Poisson distribution (Adler and Hasofer, 1981, Theorem 6.9.3, p. 1611). According to the ‘Poisson clumping heuristic’, high maxima of an SPM are distributed over space according to a Poisson process. This result is used to control family-wise error in SPMs, with the aim of identifying surprisingly high or broad excursions. We will use it here to a different end.

Other methods have been put forward to provide meta-analysis of spatially distributed local maxima/peaks (Wager et al. (2009) and Eickhoff et al. (2009)). These approaches convolve observed peak locations with a kernel and examine overlap between studies. Our method replaces the free parameter of kernel width with a pre-defined parcellation of the search space into scientifically meaningful brain regions. Under different assumptions, both Thirion et al. (2007) and Xu et al. (2009) both provide useful methods for inferring inter-subject effects. Our approach differs in the following way. The basic idea we pursue is simple: if the information in an SPM is contained in the local density of peaks, the variations of this density can be detected by simple counting statistics. This means we can identify experimentally-induced changes in spatial patterning over a partition or set of pre-specified regions. Here the spatial distribution of supra-threshold events can be earmarked as surprising, even when the existence of each event is not. The method is not concerned with the absolute number, the height or spatial extent of individual activations: It addresses only the spatial distribution of events over the partition as a multivariate summary statistic. The proposed approach provides a control on the relative density of ‘hits’ across the brain volume: if (say) all the brain is active, the proposed method will report regions that are more active. Thus the regional specificity control allowed by the proposed approach is a relative control.

In what follows we first introduce some definitions and define the Poisson Process upon which inference is based. We then relate these to the task of refuting chance patterning of peaks in one or more SPMs. We then show that the approach has acceptably low error using simulated data. Finally, we use real data to show that the method can be more powerful (identify more regionally specific responses) than conventional analyses using RFT.

## Theory

### Set-up and preliminaries

Let an SPM be defined on *A* ⊆ ℜ^*D*^. Now partition *A* into regions, *A* = {*A*_*j*_}_*j*∊1,…,*n*_ and let *d*_*j*_ indicate the integer number of events in *A*_*i*_. Under any (e.g. non-isotropic) null SPM, this number depends on the ‘statistical volume’ of *A*_*i*_ which we denote |*A*_*j*_|..For an isotropic SPM, |*A*_*j*_| is directly proportional to the physical volume of *A*_*j*_. Otherwise, the measure of the *j*-th region |*A*_*j*_| is its RESEL count and is estimated easily using conventional techniques (Worsley et al., 1999; Taylor and Worsley, 2007). See Appendix 1 for details.

### The Poisson clumping heuristic

A point-process over *A* is a homogenous Poisson process if and only if the joint distribution *d* = [*d*_1_, …*d*_*n*_] over any fixed partition *A* = {*A*_1_, ..., *A*_*n*_} is a mutually-independent Poisson-distributed random vector with emission rate *λ* > 0; i.e. *d*_*j*_ ∼ Poisson(*λ*|*A*_*j*_|). For our purposes, the crucial property of a homogenous Poisson process is that events fall uniformly and independently over space (see Appendix 2). As a consequence, if we are given the total event count, *d*_1_ + ... + *d*_*n*_ = *k*, the joint distribution of event counts is multinomial

(1) $$\begin{array}{c} p\left(d|a\right)=\frac{k}{d_{1}!d_{2}!\ldots d_{n}!}\prod_{j=1}^{n}a_{j}^{d_{j}}\\ a_{j}=\frac{\left|A_{j}\right|}{\left|A\right|}\text{.} \end{array}$$

In resel-space, the spatial deployment of maxima follows a homogenous Poisson process (see Taylor and Worsley, 2007). We therefore make heavy use of Eq. (1) as a likelihood model for spatial patterning over a pre-specified partition of the SPM. With a likelihood model, which embodies our expectations about spatial patterning under the null, we can now disambiguate observed spatial inhomogeneity as arising from noise versus signal.

### The likelihood model

Consider two models *M*_*i*_:*i* ∊ 0, 1, which postulate null uniform and alternative non-uniform spatial patterns of events. *M*_0_ supposes that the expected proportion of events falling in region *A*_*j*_ is simply the relative regional resel count *a*_*j*_:∑*a*_*j*_ = 1. In contrast, *M*_1_ allows experimentally-induced departures from uniformity; the expected proportion of events in *A*_*j*_ can be anything and is denoted by *θ*_*j*_:∑*θ*_*j*_ = 1. Here the vector *θ* = [*θ*_1_, …*θ*_*n*_] describes the probability that a given event will fall in each of the *n* regions. The strategy of this paper is to represent and update beliefs about *θ*. Before proceeding with a Bayesian treatment, we comment in passing on classical inference using this model:

Classical decision schemes to reject the null-patterning

(2) $$\begin{array}{l} H_{0}:\theta =a\\ H_{1}:\theta \neq a \end{array}$$

are easy to implement using the *χ*^2^ test of multinomial outcomes. This requires a moderate number of events in each region, which can be assured using a low threshold or regions with large resel counts. An interesting special case is the bipartition *A* = {*A*_1_,*A*_2_}. Imagine that some small, pre-specified region is of interest and the complement of this region (the rest of the brain) completes the partition. Knowing that there are *k* supra-threshold events in the whole brain, *d*_1_ of which observed in the small volume, we can easily calculate classical ‘*p*-values’ for the observed pattern, under the null:

(3) $$p\left(d|H_{0}\right)=\sum_{i=d_{1}}^{k}Binomial\left(i;k,a_{1}\right)\text{.}$$

Extensive simulations (not reported here) reveal this *p*-value tends to be slightly less conservative than set-level inference (Friston et al., 1996) on the small volume *A*_1_. Set-level *p*-values are based on random field theory and report the probability of observing a given number of ‘events’ *k*, above some pre-specified height and size threshold in a volume of measure |*A*|. We now turn to the Bayesian inference, which allows us to develop hierarchical models with a wider domain of application.

### Bayesian inference

We are initially agnostic about the null and alternative models $p\left(M_{i}\right)=\frac{1}{2}:i\in 0,1$ and use Bayes theorem to update their relative credibility *a posteriori* to observing the SPM. Here, *p*(*M*_*i*_) is the *a priori* belief that *M*_*i*_ is the correct model and the posterior is:

(4) $$p\left(M_{i}|d\right)=\frac{p\left(d|M_{i}\right)p\left(M_{i}\right)}{\sum_{i}p\left(M_{i},d\right)}\text{.}$$

This update requires the integrated likelihood or evidence:

(5) $$p\left(d|M_{i}\right)=\int p\left(\theta ,d|M_{i}\right)d\theta =\int p\left(d|\theta \right)p\left(\theta |M_{i}\right)d\theta$$

which penalises complexity and ensures that *M*_1_ is not unfairly advantaged (e.g. Rasmussen and Ghahramani, 2000 — and see below). Under the Poisson clumping heuristic, we take the likelihood to be (cf Eq. (1)):

(6) $$p\left(d|\theta \right)=k\prod_{j=1}^{n}\frac{\theta_{j}^{d_{j}}}{d_{j}!}\text{.}$$

And the priors are determined by the model:

(7) $$\begin{array}{l} p(\theta |M_{0})=\delta (a)\\ p(\theta |M_{1})=Dir(cm)\text{.} \end{array}$$

Here *δ*(⋅) is a degenerate distribution, zero everywhere but for its argument. This specialises Eq. (5) to Eq. (1). *Dir*(*cm*) denotes the Dirichlet prior on *θ* where *m* gives the prior mean and *c* relates to the prior precision (see below for more details on the Dirichlet). In the present context we specify an uninformative Jeffries Dirichlet prior in which each element of this *n*-vector is set equal to 1/2 (Jeffreys, 1946, 1961). This uninformative Dirichlet does not favour any particular pattern over any other in contrast to the informed null model, which assumes one pattern (i.e. *θ* = *a*). These models therefore have different implications for predicted observations *d* (Eq. (5): the ‘prior's prediction’). Intuitively, the uninformative prior distributes probability mass diffusely over the set of possible patterns. By contrast, the informative prior apportions high probability to any observed data pattern *d* close to *a* at the expense of patterns far away. This is the mechanism behind [Bayesian] Occam's Razor: informed models are less surprised by data near *a*, relative to data that deviate from *a*.

To see this concretely, consider a bipartition (e.g., over brain hemispheres) and two mean-*a* priors, one with highly concentrated prior mass *c* = *∞* (i.e., the null model *M*_0_) and one with lower concentration *c* ≪ *∞*. It can be shown that under our priors, the dispersion of the distribution given in Eq. (5) is:

(8) $$\mathrm{var}\left(d|a,c\right)=ka(1-a)\left(1+\frac{k-1}{c+1}\right)\text{.}$$

The first (null) model thus has competitive advantage over the alternative (i.e. attributes higher probability in Eq. (5)) for any data consistent with the prior expectation *a*. This implements Occam's razor. In this example, model selection reduces to comparing two distributions with equal means and different variances, so that the null model is preferred when the data fits the model, while the converse model is preferred otherwise. A more general result for the predictive variance–covariance of the Dirichlet-multinomial is given in Tripsiannis et al. (2002).

This scheme provides a simple way to make inferences on models and test hypotheses. Model evidence however is a gross measure: strong evidence for non-uniform patterning can arise from an excess of events in just one (or more) region of the partition. The success of the alternate model, as judged by its higher evidence (i.e. a large Bayes factor) can further be explained by examining the posterior density on its parameters: *p*(*θ*|*d*,*M*_1_) ∝ *p*(*d*|*θ*,*M*_1_)*p*(*θ*|*M*_1_), which encode each region's relative propensity to emit an ‘event’. Inference on regional parameters can be finessed with appropriate adjustments to posterior confidence, if we infer on a large number of parameters (see below). In what follows, we consider Bayesian inference on single SPMs and multiple SPMs acquired from different subjects under the same conditions.

### Multi-subject models

So far, we have only considered inference on a single SPM (e.g. from one subject). The strategy for multi-subject analyses depends on one's belief about between-subject variation. If all between-subject variation in spatial patterning arises from noise (i.e. the form of any structured inhomogeneity is conserved over subjects), a fixed-effects strategy is appropriate. This motivates pooling of data (i.e., regional event counts) over subjects, because the subject index is not informative of true variation. Alternatively, if we believe there is true inter-subject variation in regional patterning, one can pursue a random-effects (RFX) strategy. In this case, pooling must be more qualified: subject indices carry information about true variation and inference focuses on the population mean. We first generalise the formulation above to accommodate subject-specific indices. We then describe two schemes that are suitable in the fixed and random-effects cases.

Let *A*_*i*_ = {*A*_*i*1_, ..., *A*_*in*_}:*i* = 1, …,*I* now represent the partition of the *i*-th subject. As above, under *M*_0_, the *A*_*ij*_ govern the probability that a given event in the *i*-th subject will fall in region *j* or, equivalently, the expected fraction of events falling in region *j*. Under *M*_1_, the corresponding probability is *θ*_*ij*_ : ∑ _*j*_*θ*_*ij*_ = 1; the vector *θ*_*i*_ now describes, for the *i-*th subject, the probability of a given event falling in each of the *j* = 1,…,*n* regions, assuming there is experimentally-induced patterning. Notationally, the *i*-th subject data now yields data *d*_*i*_. Henceforth, we redefine *d* = [*d*_1_, ...., *d*_*n*_] to denote the entire data-set over subjects.

### Fixed-effects models

Under fixed effects (FFX), subject-specific indices are uninformative regarding putative patterning and can be ignored. Pooling over uninformative subject-specific indices, we can define *A*_*j*_ = ∪ _*i*_*A*_*ij*_. Inference on this ‘hyper-subject’ now reduces to the scheme described above, by simply pooling regional resel and event counts over subjects. (The values *d*_*ij*_ are summed over *j* to yield a ‘summation subject’, in which between-region effects can be detected.)

### Random-effects models

If we believe that there is real between-subject variation in patterning, we can use our random sample of subjects to infer on the population from which they came. It is convenient to assume a parametric form for the population. Here, we assume they are distributed according to a Dirichlet, whose parameters we aim to infer. The parameter vector *θ*_*i*_ = [*θ*_*i*1_, …, *θ*_*in*_], which characterises the pattern of the *i*-th subject is therefore sampled from:

(9) $$\begin{array}{c} p\left(\theta_{i}|c,m\right)=\frac{\Gamma \left(\sum_{i=1}^{n}cm_{j}\right)}{\prod_{j=1}^{n}\Gamma \left(cm_{j}\right)}\left(\prod_{j=1}^{n-1}\theta_{\mathrm{ij}}^{cm_{j}-1}\right){\left(1-\sum_{j=1}^{n-1}\theta_{\mathrm{ij}}\right)}^{cm_{n}-1}\\ m_{j}=E\left(\theta_{\mathrm{ij}}\right):\sum m_{j}=1\\ \sum_{j=1}^{n}\theta_{\mathrm{ij}}=1:\forall i\text{.} \end{array}$$

Where Γ is the gamma function and *θ*_*ij*_ > 0:∀*i*, *j*. The components of *m* = [*m*_1_, …, *m*_*n*_] define the proportion of events in each region, expected over subjects in the population. Here, *m*_*j*_ is the regional population mean we seek, around which subjects vary according to a Dirichlet whose variance is controlled by *c* > 0. Again, if there was no systematic, experimentally-induced spatial patterning at the group level, *m* = *a* is simply equal to the relative resel counts, by the preceding arguments. For Bayesian inference on these quantities, we represent our *a priori* uncertainty about the population parameters with *p*(*m*,*c*).

Model comparison is more problematic in the random-effects case. In particular, the integrated likelihood has no analytic solution. For convenience, we restrict inference to the population means *m*_*j*_ rather than models *M*_*i*_. As we shall see, this does not pose an obstacle to useful inference at the population level. Under RFX models, we can ask whether, on average, individuals deviate from null-patterning, for any region as follows. First, we consider the set of marginal distributions *p*(*m*_*j*_|*d*) = ∫ *p*(*m*,*c*|*d*)*dcdm*_∼ *j*_, where *m*_∼ *j*_ is the vector of population means, except for region *j*. We can obtain a stochastic approximation to this integral (to arbitrary precision) via well-understood methods (see Appendix 3). From these we can derive confidence intervals; e.g. *CS*_95_(*m*_*j*_) that can be penalised for multiple inferences, as described next. This permits us to identify regions with an unusual density of events. We note that classical RFX analysis, under the same Dirichlet assumptions about the population, may be achieved via frequentist results found in Paul et al. (2005).

### Inference on regional parameters

When making separate inferences about regional parameters, we encounter a multiple comparisons problem, if we use a high posterior confidence that *m*_*j*_ ≠ *a*_*j*_ (or *θ*_*j*_ ≠ *a*_*j*_) to declare a region ‘significant’. Note when we compare models there is no multiplicity problem. There is only one model comparison and the integrated likelihood is automatically penalised in relation to the number of free regional parameters. From one perspective, parameters play an auxiliary role in quantifying why the null pattern has been rejected by model comparison; in this view, parameter inference *per se* is unnecessary. However, from the perspective of inference on parameters (under a selected model), it may be desirable to seek some form of control at the parameter level if each parameter is reported in relation to its marginal posterior. This is particularly important if no omnibus test (e.g., Bayes factor) is available and there are many parameters.

With this in mind, recall that an ‘*x*% Bayesian confidence interval’ summarises where *x*% of our posterior belief in the true parameter lies. Under our RFX model, the posteriors *p*(*m*|*d*) and *p*(*m*_*j*_|*d*) are unimodal (the Dirichlet distribution is a convex function of *m* because it is in the exponential family); this also applies to our FFX model. Therefore, we use central confidence intervals for both FFX and RFX models. With two regions (with one degree of freedom because regional parameters must sum to one), these confidence intervals exclude extreme tails attributed with *ε* = (1 − *x*) net credibility. Consequently, we choose to penalise (increase) confidence intervals according to the number of regions minus one by enforcing *ε* = (1 − *x*)/(*n* − 1). We do not want to justify a Bayesian approach in terms of frequentist error control, which would be inappropriate. However, as we will show, under the conditions of our simulation, our approach incidentally provides frequentist control of false detections.Unless otherwise stated, we use *x* = 99%.

### A note on informed models

In the preceding sections, we described schemes for evaluating the mismatch between an observed pattern and that estimated under vague prior assumptions. In some situations, one may have a precise alternative model or hypothesis. This could be derived from the spatial profile *m*_1_ observed in an independently replicated experiment. Precise or informed alternative models are easily accommodated in the FFX analysis by substituting a degenerate Dirac delta for the prior: *p*(*θ*|*M*_1_) = *δ*(*m*_1_) (assuming high confidence about *m*_1_). With RFX models, precise priors *p*(*θ*|*m*,*M*_1_) = *δ*(*m*_1_) finesse the complications in evaluating the integrated likelihood and enable straightforward RFX model comparison: having specified *m* under the null and alternative hypotheses, the model evidence obtains by integrating the likelihood with respect to the population means (trivial by exploiting Dirichlet-multinomial conjugacy) and the scalar *c* (integrated with any simple numerical scheme). Note, by definition, the parameters of an informed model (such as the null) are specified by the hypothesis. There is therefore no component-wise inference on their parameters; inference is between two hypothetical patterns. We will explore the applications of informed model comparison in future work.

## Simulations

### Frequency evaluations

Bayesian error control, imposed by integrating the data-likelihood under vague priors, does not aim to satisfy Frequentist criteria (e.g. control the false-positive rate). It is nevertheless interesting to examine how strongly the results depend on the inferential scheme. In this section, we simulate experimental data and evaluate the Frequentist behaviour of our Bayesian scheme. We explore this behaviour in the absence of experimentally-induced patterning, by generating data with no signal. For each of 84 volumes in the simulated experiment, independent unit-variance Gaussian noise was introduced onto a 64 × 64 × 64 regular lattice. We induced non-stationarity with piecewise constant smoothing over twenty random regions; obtained through a Voronoi parcellation diagram with random seeds (Flandin et al., 2002). Each region was smoothed independently with its own full width half maximum FWHM drawn uniformly on the interval [4, 10] mm. To preclude sharp transitions at regional boundaries, we then smoothed the entire image again with a Gaussian kernel (FWHM of 2 mm). This was repeated 1000 times to create surrogate data under the null hypothesis. We then fitted GLMs with a simple mean effect and collected the ensuing *t*-fields or SPMs.

We randomly sampled *L* = 150 groups of 20 simulated subjects from our corpus. For each of these simulated experiments and ensuing SPMs, we defined ‘regions of interest’ by randomly selecting a fixed number of *N* regions from the AAL anatomical parcellation scheme (*Tzourio-Mazoyer et al., 2002*). *N* ∊ {5,10,15,20}. We used the union of the remaining regions in the AAL parcellation (i.e., the rest of the brain) as our final ‘region’. We calculated the regional resel counts of each ‘region’ and the corresponding number of events above a height threshold of three. We then assessed the Frequentist properties of fixed- and random-effects inference.

### Fixed effects

All rational decisions (e.g. ‘tests’) require some subjective notion of utility/loss. Conventional decision thresholds are somewhat arbitrary (e.g. set such that alpha = 0.05). A threshold of 20 is the convention for Bayesian decisions based on relative evidence (given a Bayes factor). To begin, we defined a Bayes factor of twenty (i.e., very strong evidence) as the threshold for accepting the alternative model. We found that no Bayes factor from any of the simulated groups attained this threshold for any *N* ∊ {5,10,15,20} regions. This indicates that under our decision threshold and the conditions of our simulation, our Bayesian procedure incidentally implies a low false-positive rate in terms of model selection.

For inference on parameters, we estimated of the Frequentist Family-Wise Error Rate (FWER) using $P(V\geq 1)=E(I(N))\approx \frac{1}{L}\sum_{l=1}^{L}I_{l}(N)$. Here *I*(*N*), *I*_*l*_(*N*), ..., *I*_*L*_(*N*) are identically distributed: the approximate equality $E(I(N))\approx \frac{1}{L}\sum_{l=1}^{L}I_{l}(N)$ says that expectation of interest is approximated by the empirical average over *L* realised observations. Here the random variable *V* denotes the expected number of frequentist errors in an experiment, the indicator *I*(*N*) = 1:*V* > 0 (i.e. with one or more regions whose penalised confidence intervals were inconsistent with the null) and 0 otherwise. *L* is the number of simulated replications over which we take the empirical expectation. We observed no FWER greater than 0.05 for any *N* ∊ {5,10,15,20} regions. FWER on regional parameters were {0.013, 0.013, 0.046, 0.033} respectively. These findings indicate that our procedure incidentally limits false-positive decisions on regional parameters to a small rate.

### Random effects

In the context of random-effects models, we restrict our analysis to inferring parameters (not models). We used the RFX scheme to infer regional parameters for the same set of null experiments. Again, for each experiment, we counted the number of SPMs with one or more parameters, whose penalised confidence intervals were inconsistent with the null. We observed no FWER greater than 0.05, for any *N* ∊ {5,10,15,20} regions: the observed values were {0.006, 0.000, 0.043, 0.043} respectively.

### Supplementary analyses of parcellation and data smoothing

The preceding validations consider partitions with a relatively small number of areas. In some situations, we may have no priors on the functional anatomy and prefer a more exploratory approach. It is easy to validate the FFX procedure for many regions. We performed the same simulation procedure described above, but with *N* = 116; i.e., the entire set of regions in the AAL. Our empirical estimate of the FWER on regional parameters was 0.04, validating the method for open-ended exploratory use.

Strictly speaking, data smoothing should have no effect on the robustness of the scheme because we effectively work in RESEL space (where the effects of smoothness are removed). More precisely, our null model conditions on the RESEL count associated with each region. This means the model does not depend on the degree of smoothing. Nevertheless, smoothness will affect the production of maxima in each individual SPM and therefore affect regional counting statistics. To illustrate that the approach is robust to different levels of data smoothing, we simulated 1000 SPMs using data smoothed with an 8 mm Gaussian kernel. Using the above procedure and under the conditions of this simulation, we found a regional FWE of 0.003 and no false model comparisons.

## Applications: the regional anatomy of language

### Motivation

A reliable measure of language laterality is important for both basic and clinical research. Furthermore, lateralisation represents a canonical example of a functional pattern one might want to make inferences about. Language laterality in fMRI is usually assessed by computing a Laterality Index (LI) that compares the relative contribution of both hemispheres, during a given language task. However, several methodological issues may confound the LI in healthy and diseased populations; for a critical review see Seghier (2008). All previous studies have assessed LI values based on either extent (e.g. size of left or right activated regions that survived a pre-defined threshold) or height (e.g. grouping statistical scores within a region of interest). Our pattern perspective aims to infer the spatial profile of local maxima, bypassing inference on extent or height of local activations. This perspective may be more fitting, particularly for laterality measures, in that it assesses the relative spatial distribution of events. Laterality is a rather coarse characterisation of functional localization. We therefore proceed to ask which specific language areas, within a more fine-grained parcellation of the brain, show laterality effects.

### The task and data

We demonstrate our method on a data-set from previous work (Seghier and Price, 2009). These data were obtained from 24 healthy subjects (9 males, 15 females, age: 36 ± 18 years). All subjects were native English speakers, had normal or corrected-to-normal vision, with no history of neurological or psychiatric disorders; and were right-handed as assessed with the Edinburgh questionnaire (Oldfield, 1971). Over the block paradigm design experiment there were 16 blocks presenting written object names and 8 blocks presenting strings of unfamiliar Greek symbols, each lasting 18.8 s with an additional 12 blocks of 14.4 s fixation every two stimulus blocks. All stimuli were presented as triads (three visual stimuli, with one target above and two choices below). Subjects were asked to press a button indicating whether; (i) the stimulus on the lower-left or lower-right was more semantically related to the target above (e.g. is ‘truck’ or ‘ship’ most closely related to ‘anchor’) or (ii) the unfamiliar symbols on the lower-left or lower-right were visually identical to the target. All subjects performed well on this matching task (performance = 92 ± 7%).

Data were acquired on a 1.5 T Siemens system (Siemens Medical Systems, Erlangen, Germany). Functional imaging used an EPI GRE sequence (TR = 3600 ms, TE = 50 ms, Flip = 90°, FOV = 192 mm, matrix = 64 × 64, 40 axial slices with 3 × 3 × 3 mm voxel size). Data processing and statistical analyses were carried out with the Statistical Parametric Mapping SPM5 software package (Wellcome Trust Centre for Neuroimaging, London, UK, http://www.fil.ion.ucl.ac.uk/spm/). All functional volumes were spatially realigned, un-warped, normalised to the MNI space, and smoothed with an isotropic 6-mm FWHM Gaussian kernel, with a resulting voxel size of 2 × 2 × 2 mm. The pre-processed functional volumes for each subject were then submitted to a conventional fixed-effects SPM analysis, using a general linear model at each voxel. Each stimulus onset (except fixation) was modelled as an event encoded in condition-specific ‘stick-functions’. The resulting stimulus functions were convolved with a canonical hemodynamic response function to form regressors for the linear model. Our contrast of interest was the main effect of semantic matching on words, relative to perceptual matching on unfamiliar symbols. More details about this analysis and the main effect of interest during semantic matching can be found elsewhere (see Seghier and Price, 2009). Our task is used routinely in clinics and reliably identifies language areas and functional laterality (e.g. Seghier et al., 2004).

### Data analysis

Our FFX model can be thought of as a limiting case of the RFX model. In particular, as the between-subject variability decreases, subject-specific spatial patterns fluctuate around a single ‘fixed’ value. We therefore deliberately chose a data-set with relatively low between-subject variability, so that we can plausibly consider both FFX and RFX models on the same data. To ensure this homogeneity, we conditioned the sample on right-handedness, a covariate known to induce important between-subject variability (Pujol et al., 1999; Szaflarski et al., 2002). As we shall see, the inferences under RFX and FFX were very similar. In general, the latter expects, and accounts for, more sources of variability. Therefore posterior inference is less confident: i.e., it returns a smaller set of ‘significant’ regions. For all the analyses below, we counted peaks above a height threshold of *t* = 3. These event counts served as the data-features of interest.

### Fixed effects

We began by defining two regions for the entire right and left hemispheres; excluding the cerebellum due to the crossed cerebellar representation of laterality; and the mesial cortex near to inter-hemispheric fissure (for more details see Seghier, 2008). Using this hemispheric partition we obtained a Bayes factor of 2 × 10^11^ in favour of language lateralisation. At the level of parameters, we found that the confidence interval for the average proportion of ‘events’ in the left hemisphere, *CI*(*θ*_1_) = [0.525, 0.553], was inconsistent with, that expected by chance 0.4962; i.e. that based on resel counts. On applying RFX analysis, we again found that the confidence interval for the estimated average fraction of events in the left hemisphere, *CI*(*m*_1_) = [0.521, 0.565], was inconsistent with that expected by chance alone (0.4962). From either of these we conclude that there is evidence for left-lateralization of language.

We then defined nine language-related regions in the left hemisphere, based on previous meta-analysis studies (e.g. Cabeza and Nyberg, 2000; Vigneau et al., 2006) and their homologous regions in the right hemisphere. These are shown in Table 1, in the AAL parcellation scheme (*Tzourio-Mazoyer et al., 2002*).

We used these regions to partition the brain into 19 areas (9 language bilateral regions and the remaining brain volume) to see whether we could characterise lateralisation with greater regional precision. Under FFX assumptions, we again found evidence for a non-uniform pattern, with a Bayes factor of 1.149 × 10^22^. To understand this result, we turned to the parameters. Table 2 lists those regions that were surprising, in terms of the relative number of high peaks observed using both FFX and RFX models.

Note that there is overlap between the regions returned by the both analyses. As expected, high activity tends to be in the left hemisphere and low activity tends to be in the right hemisphere.

**Fig. 1:**
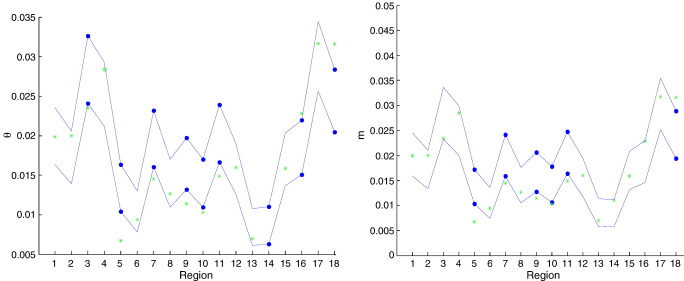
Exploratory FFX analysis assuming a parcellation that includes all areas in the AAL, except cerebellum (see text). These 28 axial slices report regions deemed relatively rich (sparse) in local peaks in red (green). In most slices, there is a relative preponderance of activations in the left hemisphere and deactivations in the right hemisphere.

To visualise the basis for these inferences about regional specificity shown in Table 2, Fig. 1 illustrates the marginal confidence intervals. These intervals pertain to the nine regions listed Table 2, bilaterally. The blues lines correspond to the corrected 99% confidence intervals for each region. (They are joined simply for ease of graphical inspection.) The green dots are the relative RESEL count for each region. The left panel corresponds to FFX analysis on *θ*, while the right panel corresponds to RFX analysis on *m*. We can see from these plots that some of the confidence intervals exclude the null (green dots). For ease of visual inspection, we highlight such regions with embolded confidence bounds (thick dots).

### Exploratory analysis

**Fig. 2:**
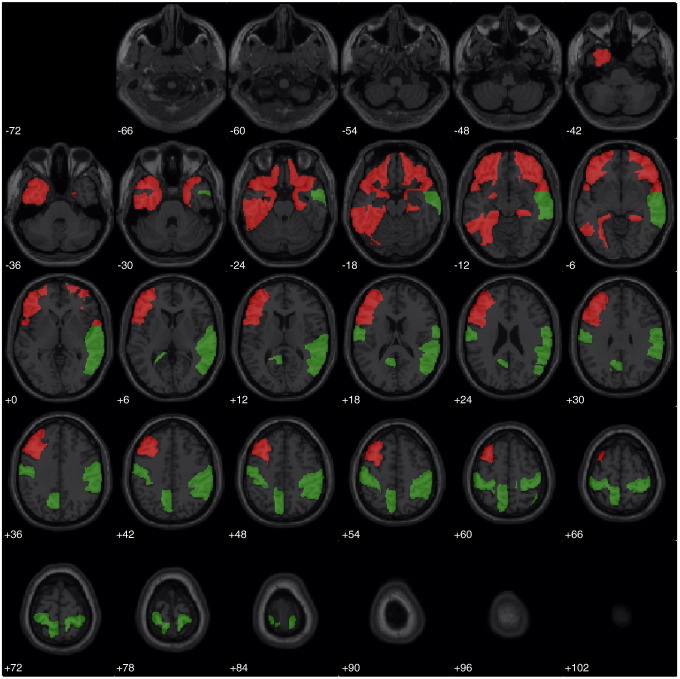
This plot gives corrected confidence bounds (blue lines) and the null (relative RESEL count) setting for each of the 18 regions assessed. For ease of inspection, we highlight — with thick solid dots — regional confidence intervals that exclude the null. The figure shows that several regions substantially deviate from null expectations. The regions are: left/right ‘Precentral’ (1,2), left/right ‘Frontal_Mid’ (3,4), left/right ‘Frontal_Inf_Oper’ (5,6), left/right ‘Frontal_Inf_Tri’ (7,8), left/right ‘Frontal_Inf_Orb’ (9,10), left/right ‘Fusiform’ (11,12), left/right ‘Angular’ (13,14), left/right ‘Temporal_Sup’ (15,16), left/right ‘Temporal_Mid’ (17,18).

For illustration, we then assumed nothing about the functional anatomy engaged by the task comparison and use a more exploratory approach, which is useful when there is little *a priori* knowledge of the functional anatomy. In particular, we choose all regions, excluding the vermis and cerebellum, as our partition. Table 3 and Fig. 2 report the results. As before, there is a preponderance of left-hemispheric regions that are relatively rich in events. Conversely, right-hemispheric regions tend to be relatively sparser in high peaks. The majority of these areas have been associated with language function in other studies (e.g. Seghier et al., 2004; Vigneau et al., 2006).

### Supplementary null and comparative analyses

As a final check on our model assumptions we analysed SPMs based on real data that conformed to the null hypothesis: if our null Poisson Process model is not tenable for real data, the fraction of events found in each region should not be approximated by the relative RESEL count. For each of 15 subjects we calculated an SPM testing for the effects of a random covariate (independent draws from the normal distribution). The results are null SPMs by construction. We repeated this procedure ten times and were never able to reject the null model, according to the decision procedures used above.

Finally, we compared the results from pattern inference with two conventional whole-brain approaches based on the height and extent of SPM excursion sets. To do this we computed an SPM of the *t*-statistic, using the 24 subject-specific contrasts of parameter estimates above. For both FWE adjusted thresholds, we catalogued all AAL regions containing at least one supra-threshold voxel. Pattern inference identified relative regional effects in five regions not identified by either conventional SPM analysis:

• ‘Frontal_Sup_Orb_L’,
• ‘Frontal_Mid_Orb_R’,
• ‘Frontal_Sup_Orb_R’,
• ‘ParaHippocampal_R’,
• ‘Temporal_Pole_Sup_R’

Conversely, FWE procedures identified regions that were active in absolute — but not relative — terms. Four regions were identified using peak height (two of which showed regionally specific effects based on pattern inference). Inference based on spatial extent identified 29 regions (of which nine showed regionally specific responses). This emphasises that inference about relative vs absolute responses are distinct. In other words, inferring that a region has responded does not necessarily mean the response is regionally specific (i.e., not more than other regions).

## Discussion

We have introduced a new method, which can identify experimentally-induced changes in spatial patterning over a set of pre-specified regions. Here, inference is on the spatial organisation of events (high peaks) rather than their absolute number or the attributes (e.g., height or extent) of individual activations. A positive decision about region Q means that region Q is relatively sparse or rich in events, *relative to the rest of the search volume*. Note that the interpretation is inherently relative. The rest of the brain may or may not be activated in absolute terms i.e. as determined by conventional peak or cluster-extent methods. It is in this sense that the method infers *patterns —* attributes of the SPM that are distributed over whole search volume. It therefore differs qualitatively from existing methods, and is complementary to them. While we have focused on patterns in SPMs of functional images, the method is clearly applicable to structural (e.g. VBM) analyses, for which there is also a clear null hypothesis (Ashburner and Friston, 2000). We have emphasised that pattern inference is on a region's response relative to average the activity of other regions. Therefore, it will not detect regions when there is a uniformly high *absolute* peak rate (e.g. as inferred via set-level inference). In such conditions, rejecting a regionally nonspecific (global uniform) hypothesis is more conservative than rejecting a (global null) hypothesis that no region has responded.

Note that the model underlying pattern inference does not, strictly speaking, assume independence of counts across regions: given the total count, components of a multinomial random vector have negative covariance. For region-wise tests, this negative dependence means that falsely inferring an event excess in one region increases the chance of inferring event dearth in the remaining regions. Our simulations indicate that the *n* − 1 penalty furnishes appropriate control, despite this dependence. Its success is not surprising, given the formal similarity of this penalty to Bonferroni-correction, which holds under arbitrary dependence.

To assess the evidence for experimentally-induced spatial patterning over a set of pre-specified regions, we must account for two confounding explanations: (i) spatial inhomogeneity in the SPM and (ii) the relative volume of cells. We exploit an established measure of ‘statistical’ volume (the resels-per-voxel image) to attain a volume measure that effectively removes local variations in the geometry of statistical dependencies, under the null. We use this in conjunction with the Poisson clumping heuristic to elaborate a hierarchical pattern model, which affords inference on both model and parameter (pattern) space; at the within or between-subject level.

As an illustration, we applied the method to ask whether a language task influences the pattern of event in the ensuing SPMs. We identified specific regions of a language network that had a surprisingly low or high proportion of events, given their volume. In particular, left-hemisphere regions tended to be relatively rich in events, and right-hemisphere regions were relatively sparse. It is noteworthy that there was close agreement between RFX and FFX inferences. This is partly because our FFX is naturally robust to *local* (within region) functional heterogeneity over subjects; it sees only the regional count. Only heterogeneity between different regions would benefit from an explicit random-effects model. Additional factors limiting functional heterogeneity (e.g. when considering functionally conserved brain systems or conditioning on relevant covariates) should bolster the suitability of FFX analyses. This is fortunate for practical reasons; the analytic FFX solution is quick, benefits from an exact model evidence, and is more suitable for exploratory (high-dimensional) analyses.

As we have demonstrated, pattern inferences can be used for hypothesis-driven as well as exploratory analyses. In the former case, the motivation for choosing a specific parcellation derives primarily from the scientific question. The approach we have illustrated started with building blocks, defined within an existing parcellation scheme (i.e., AAL), and aggregating regions when desirable. Regions can be grouped according to prior knowledge of the functional neuroanatomy. For example, one may ask whether bilateral frontal *vs* bilateral temporal regions are more engaged in a task. Here, one would distinguish lobes while pooling across hemispheres into a two-region partition (three if considering the complement of the brain). Note that each partition embodies a different (null and alternative) hypothesis. This means one can address the same SPM with different hypotheses, framed in terms of different partitions. We have tried to illustrate this anecdotally by using different partitions above, when charactering language activations. One can imagine step-down applications of this approach; where a cell from a ‘significant’ partition is itself partitioned and the process repeated recursively, until no further functional segregation can be inferred. We will pursue this in elsewhere. In a hypothesis-driven approach, partitions are informed by known functional neuroanatomy. Previous research provides *a priori*, constraints on the inference: known functional anatomy can be used to restrict the search to a small number of regions. This empowers inference, because reducing the number of regions reduces the implicit penalty imposed by the integrated likelihood or multiplicity-controlled confidence intervals.

We emphasise that our inference about spatially structured responses is no more than that (i.e., a test of departure from the null hypothesis of uniform or non-segregated effects). The regional specificity of this inference is determined by the nature of the partition. The partition can have a small number of large regions (e.g., right vs. left hemisphere); in which, case the inference will have little regional specificity but good sensitivity to effects that are not conserved spatially over subjects. Conversely, the partition can have a large number of small cells, in which case the inference and *post-hoc* interpretation of the pattern will be regionally specific. Sensitivity here depends on any responses being expressed within the same cells. It is interesting to think about the limiting case in which the partition includes the set of all voxels and how this relates to standard topological inference. In a subsequent paper, we will look at optimising the partition with respect to sensitivity and the implicit dependence on the spatial scale at which activations are conserved over subjects.

Beyond the effects of the spatial prior, implicit in the partition, the quantity of data available may also influence the choice of partition. Information about regional parameters will increase with the number of data-features, and hence with the number of subjects and size of the regions. Additionally, as applied to peaks of an SPM, our proposed scheme also depends on two pre-processing specifications, which influence the number of data-features (events). First, the number of events depends (inversely) on the degree of spatial smoothing. In this respect, our method is most powerful with relatively low smoothing, e.g. FWHM of 2–3 times the voxel size (cf the pattern classification approach in Hassabis et al., 2009). Second, these features depend on a height threshold, which local peaks much transcend in order to qualify as ‘events’. Attempts to finesse this dependence have been made when inferring cluster-extent (Smith and Nichols, 2009). We have resolved this by choosing a low threshold; in this work we use *t* = 3. This is roughly the lower bound required for valid set-level inference (Friston et al., 1996). Recall that our proposed method uses (supra-threshold) peak location but not height. For very low thresholds, the fraction of events arising from the noise process alone will increase. For very high thresholds, the absolute number of events will become small. In either case, sensitivity will be compromised. Note that chance excursions above a higher threshold will have one local maxima per blob, so the position of the peak is a reasonable summary of spatial location. At lower thresholds, there may be multiple local maxima per blob (clustered closely in space). Here it is less clear how to spatially index the excursion, and spatial independence may be less tenable. In general one may resolve this by defining an ‘event’ as the highest local peak in an excursion.

The assumption that fixed-effects models are more appropriate than models that allow for random effects over subjects is clearly questionable in many contexts. Generally, fixed-effect assumptions will tighten the confidence intervals on the model's parameters, boosting the significance of the results. This is important in the current setting, because fixed-effects analyses are only tenable when between-subject variations in the expression of responses fall within — rather than between — regions of the partition. Future work will develop tools which require much weaker assumptions; i.e., non-parametric random effects — when this assumption is not tenable. Such models may provide benchmarks for justifying simpler models and enable formal model selection.

We have presented the simplest possible examples from a rich class of spatial patterning methods, which seek to understand patterns over pre-specified partitions of the brain. In future work we will describe another parametric patterning method for inferring the influence of subject-level covariates on spatial patterning. In a second line, we will elaborate on flexible non-parametric models for characterising patterned data.

## Figures and Tables

**Table 1:** Language regions, over which we considered the patterning of events.

Region	Abbreviated name
Precentral gyrus	‘Precentral’
Middle frontal gyrus F2	‘Frontal_Mid’
Inferior frontal gyrus, opercular part F3OP	‘Frontal_Inf_Oper’
Inferior frontal gyrus, triangular part	‘Frontal_Inf_Tri’
Inferior frontal gyrus, orbital part	‘Frontal_Inf_Orb’
Fusiform gyrus	‘Fusiform’
Angular gyrus AG	‘Angular’
Superior temporal gyrus	‘Temporal_Sup’
Middle temporal gyrus T2	‘Temporal_Mid’

**Table 2:** This table reports which regions, from those outlined in Table 1, were surprisingly short of events or surprisingly rich in events using fixed and random-effects models. In this, and subsequent tables, we only report regions whose regional parameter was greater than expected by chance (with 99% posterior confidence).

	Relatively sparse in peaks	Relatively rich in peaks
FFX	‘Angular_R’	‘Frontal_Mid_L’ ‘Frontal_Inf_Oper_L’
	‘Temporal_Sup_R’	‘Frontal_Inf_Tri_L’
	‘Temporal_Mid_R’	‘Frontal_Inf_Orb_L’
		‘Frontal_Inf_Orb_R’
		‘Fusiform_L’
RFX	‘Temporal_Mid_R’	‘Frontal_Inf_Oper_L’
		‘Frontal_Inf_Tri_L’
		‘Frontal_Inf_Orb_L’
		‘Frontal_Inf_Orb_R’
		‘Fusiform_L’

**Table 3:** Surprising regions identified by an exploratory analysis.

	Relatively sparse in peaks	Relatively rich in peaks
FFX	‘Postcentral_L’	‘Frontal_Sup_Orb_L’
	‘Postcentral_R’	‘Frontal_Sup_Orb_R’
	‘Parietal_Inf_R’	‘Frontal_Mid_L’
	‘SupraMarginal_R’	‘Frontal_Mid_Orb_L’
	‘Precuneus_L’	‘Frontal_Mid_Orb_R’
	‘Temporal_Sup_R’	‘Frontal_Inf_Oper_L’
	‘Temporal_Mid_R’	‘Frontal_Inf_Tri_L’
		‘Frontal_Inf_Orb_L’
		‘Frontal_Inf_Orb_R’
		‘ParaHippocampal_L’
		‘ParaHippocampal_R’
		‘Amygdala_L’
		‘Fusiform_L’
		‘Temporal_Pole_Sup_L’
		‘Temporal_Pole_Sup_R’
		‘Temporal_Pole_Mid_L’
		‘Temporal_Inf_L’

## Appendix 1

*Resel counts*: A resel or ‘resolution element’ is a measure of ‘statistical’ volume that generalises the Lipschitz–Killing curvature and is invariant under different spatial dependencies among the component fields (Worsley and Taylor, 2007). Following Worsley et al. (1999), we use the estimate

(A1.1) $$\left|A_{j}\right|=\int_{s\in S_{j}}RPV(s)ds$$

(or the equivalent sum for discretised images; e.g., *finite* voxel lattice approximations; see Kilner and Friston, 2010 for details). The scalar image RPV:*S* → ℜ measures ‘resels per voxel’ and is based on the covariance matrix of spatial derivatives estimated from the normalised residuals of the GLM at each point *s* ∈∊ *S*. Eq. (A1.1) just says that the effective volume in an SPM is not simply the number of voxels but the number of resolution elements. For readers who are not familiar with the formalism of SPM and random field theory, RPV can be thought of as an image which encodes the spatial roughness of underlying random terms. A voxel with a large number of resels per voxel contributes more to the statistical volume because it has the potential to emit more events. The RPV image is based on standard (if deep) results from random field theory (see Taylor and Worsley, 2007 for a technical summary).

Two related estimators of RPV(*s*) are described in (Kiebel et al., 1999) and (Flitney and Jenkinson, 2000). We use the former, as implemented in the SPM software. This computation measures roughness in terms of the variance or dispersion of the spatial differences among neighbouring voxels. It is important that we have reasonably accurate estimates of statistical volume |*A*_*j*_|, to ensure the expected number of events under the null hypothesis is estimated properly (i.e. as a benchmark against which to assess experimentally-induced patterns). Strictly speaking, the estimates are random but with sufficient degrees of freedom in the GLM, they can be treated as known quantities.

## Appendix 2

*If we have a fixed bipartition A* = {*A*_1_, *A*_2_} *and are told that there has been one local maxima, N*(*A*) = 1*, then p*(*N*(*A*_1_) = 1|*N*(*A*) = 1) = *a*_1_*.*

Proof:

(A2.1) $$\begin{array}{c} p\left(N\left(A_{1}\right)=1|N\left(A\right)=1\right)=\frac{p\left(N\left(A_{1}\right)=1,N\left(A\right)=1\right)}{N\left(A\right)=1}\\ =\frac{p\left(N\left(A_{1}\right)=1,N\left(A\cap A_{1}^{C}\right)=0\right)}{N\left(A\right)=1}\\ =\frac{\lambda \left|A_{1}\right|e^{-\lambda \left|A_{1}\right|}e^{-\lambda \left|A\cap A_{1}^{C}\right|}}{\lambda \left|A\right|e^{-\lambda \left|A\right|}}=\frac{\left|A_{1}\right|}{\left|A\right|}=a_{1} \end{array}$$

The corresponding result in the case of *N*(*A*) = *k* is binomial

(A2.2) $$p\left(d_{j}\right)=Binomial\left(d_{j};k,a_{1}\right)\text{.}$$

A multinomial likelihood arises as a natural generalisation of the above.

## Appendix 3

For computational convenience, we work with the transformed variables (*cm*) → *α* = *cm*: i.e. *c* = ∑ _*j*_*α*_*j*_ and $m_{j}=\alpha_{j}{\left(\sum_{j}\alpha_{j}\right)}^{-1}$. We exploited prior-likelihood conjugacy to integrate the complete likelihood with respect to *θ*_*i*_ — the multinomial parameters — leaving a dependence of hyperparameters *α* on the data *d*.

(A3.1) $$p(d,\alpha )=\int_{\theta }\left(\prod_{i=1}^{I}p\left(d_{i}|\theta_{i}\right)p\left(\theta_{i}|\alpha \right)\right)d\theta$$

Rather than optimising *α* (i.e. ‘empirical’ Bayes), we defined placed an independent ‘uninformative’ exponential prior on each component of the vector *α* governed by a rate of 0.01 — exp(*α*|0.01) — and drew samples from its posterior *p*(*α*|*d*). Specifically, we allowed a Metropolis Hastings process on *p*(*α*|*d*) ∝ *p*(*d*|*α*)*p*(*α*) to reach equilibrium over one million iterations. Note that with enough subjects, inferences do not depend on the particular choice of prior (Gelman et al., 2004 p 547).

We then exploited the relation $c=\sum_{j}\alpha_{j}$ and $m_{j}=\alpha_{j}{\left(\sum_{j}\alpha_{j}\right)}^{-1}$to acquire posterior samples of *m*(via deterministic transform of the sampled *α*).

The Markov dynamics were governed by an independent Gamma-distributed transition Kernel parameterised in terms of Gamma mean and variance *μ* = **a** × **b**; *σ*^2^ = **a** × **b**^2^ (where **a**, **b** are the Gamma shape and scale parameters). Thus, the proposed location of the process at each time-point in the algorithm's evolution was *μ*. The variance parameter *σ*^2^ (central to the convergence properties of the process) was adaptively updated during a burn-in epoch.
